# Measuring the Association of Self-Perceived Physical and Social Neighborhood Environment with Health of Chinese Rural Residents

**DOI:** 10.3390/ijerph18168380

**Published:** 2021-08-08

**Authors:** Pengcheng Liu, Jing Wang, Xiaojie Wang, Wenjie Nie, Fangfang Zhen

**Affiliations:** 1 School of Economics, Qingdao University, Qingdao 266061, China; qd_lpc@qdu.edu.cn (P.L.); sdgsxy1997wj@163.com (J.W.); 2 School of Management, Ocean University of China, Qingdao 266100, China; niewenjie@stu.ouc.edu.cn; 3 College of Foreign Languages, Hebei University of Economics and Business, Shijiazhuang 050061, China; zhen_f_f@163.com

**Keywords:** physical neighborhood environment, social neighborhood environment, health status, health-related behaviors

## Abstract

(1) Objectives: Using cross-sectional datasets, we investigated whether better self-perceived physical and social neighborhood environment was associated with perceived health status and health-related behaviors among Chinese rural residents. (2) Study Design: The study was based on the 2016 China Family Panel Studies (CFPS) survey. The sample consisted of 7191 rural residents over 18 in China. (3) Methods: The article measured physical neighborhood environment from the two aspects of dwelling environment (DE) and public facilities convenience (PFC), and social neighborhood environment from public security (PS) and neighborhood relationship (NR). Associations between health status/health-related behaviors and self-perceived physical/social neighborhood environment were analyzed using multivariable logistic regression models adjusted for socio-demographic characteristics. (4) Results: The results suggested that rural residents who live in a good neighborhood environment reported having a better health status. Specifically, rural residents who reported living in good DE were less likely to have a depressive mood and poor health conditions. Those who reported good PFC were less likely to have depressive mood, poor self-rated health and chronic diseases. Rural residents who reported having good PS were less likely to have a depressive mood. Those who reported good NR were less likely to have a depressive mood, poor self-rated health, chronic diseases and obesity. Regarding neighborhood environment and health-related behaviors, the results showed that rural residents who reported good PFC were more likely to do physical exercise. Notably, the regression results of the education level variable showed that education level significantly promoted the health-related behaviors (time sleeping weekday, physical exercise and smoking) of rural residents. (5) Conclusions: This article suggested that there was strong evidence for a relationship between physical and social neighborhood environment and the general health of rural residents due to all causes. According to the conclusion of this article, in order to improve the health of rural residents, policy interventions should give priority to improving the neighborhood environment. In addition, the improvement in the degree that rural residents are exposed to education is of the same importance, which helps more rural residents to maintain good health-related behaviors.

## 1. Introduction

In recent years, it has been generally accepted that in order to be effective in promoting health status and health-related behaviors, public health interventions have to address not only individual characteristics but also the neighborhood environment [1,2]. The individual health survey has identified two broad areas of the neighborhood environment that may be related to health: (1) The physical neighborhood environment including environmental factors such as air pollution, noise pollution, garbage dumping, etc., as well as the overall situation of surrounding public facilities such as education, medical care and transportation [3,4]; (2) The social neighborhood environment mainly refers to the social communication between neighbors, social norms, neighborhood security and so on [5,6]. A safe and comfortable neighborhood environment (e.g., comfortable living space, complete supporting facilities, convenient medical and social services and good neighborhood relationship) can not only reduce the prevalence rate, but also make residents happy and do good to their physical and mental health [7]. The adverse neighborhood environment (e.g., air pollution, noise pollution, dangerous physical environment, inconvenient medical and social services and bad neighborhood relationship) can form a malignant stimulus to residents and affect the resident’s psychosocial well-being indirectly [8,9,10].

A large body of literature has focused on the health effects of the physical and social neighborhood environment. Scholars have found that the physical neighborhood environment such as the noises and environmental pollution around residences can reduce residents’ self-rated physical and mental health [11,12,13,14,15], while sound public facilities and complete medical services can make residents engage in more physical exercise and get easier access to medical assistance, further lowering the incidence of chronic diseases and improving their self-rated physical and mental health [16,17,18,19,20,21,22]. The influence of the social neighborhood environment on residents’ health has also drawn certain research attentions. Living in a safe environment, residents tended to have good behaviors and a higher sense of happiness that improved their physical and mental health [23]. As has been shown in the results from Dawson et al. [24], higher levels of NR were associated with decreased depressive disorder. Yan and Zheng [25] have found that a bad neighborhood relationship including violence and the lack of social cohesion will lead to a neighborhood pressure that causes obesity, while a positive relationship will encourage residents to interact with each other and engage in exercise. In addition, moderate sports activities and better health are closely connected with the prevention of chronic diseases [26]. People living in a neighborhood where they enjoy a good neighborhood relationship and higher cohesion that make it easier for them to get assistance when they are in need tended to have a better self-rated health condition generally [27].

Although previous studies have examined the associations between self-perceived neighborhood environment and health [28,29,30], most were conducted in other countries or using the samples of urban residents, which were different from the social, economic and political contexts of rural areas with underdeveloped facilities. For example, Schulz et al. [31] examined the contributions of neighborhood characteristics in explaining associations between neighborhood poverty and residents’ cumulative biological risk in an urban community. Ruijsbroek et al. [32] examined the influence of the neighborhood social environment (social cohesion, neighborhood attachment, social contacts) on individuals’ mental health in four European cities. Compared with urban areas, rural areas revealed frequent weaknesses in public facilities convenience, living environment and medical services in terms of the physical neighborhood environment. For example, in regard to the rural transport services, improper road network planning has weakened the rural traffic efficiency seriously. In addition, with the massive discharge and random disposal of domestic sewage and garbage, the quality of the rural ecological environment kept getting worse [33,34]. In regard to health care, rural areas showed weaknesses in the distribution of both the health services staff and medical resources. In addition, the public health facilities convenience in rural areas was generally scarce and backward, which could not meet the health service needs of rural residents. In terms of social neighborhood environment, rural areas also presented different features from urban areas. First, the problem of public security is prominent. With the advance of urbanization and industrialization, the countryside has changed from a previous closed state to a current open state, which leads to the expansion of the space for breaking the law and committing crimes. Second, the neighbors interacted with each other frequently. Different from the isolated and closed living environment in urban areas, China’s rural areas are typical acquaintance societies where the exchanges between rural neighbors are more frequent and closer. The rural residents also regard “helping each other” as the ethical obligation of the neighborhood. This ethical demand has evolved into the lifestyle and moral tradition of the Chinese rural residents.

In this context, neighborhood environments and residents’ health in areas with underdeveloped rural facilities have become worthy of study. Moreno et al. [35] examined the association between perceived neighborhood problems and type 2 diabetes among rural residents in Latinos. Luo et al. [36] examined the relationship between neighborhood environment and the decline in cognitive function among middle-aged and older individuals and whether the relationship varies between rural and urban samples. One study found that neighborhood social capital is positively correlated with rural left-behind children’s mental health [37]. However, the previous research has been scattered and fragmentary; both the scope and depth of research on this topic still needs to be expanded.

Hence, the objective of this study is to investigate whether self-perceived neighborhood environment is associated with health status and health-related behaviors among rural residents of China. This article made innovations in the following two aspects: 1. Based on the special background of Chinese rural areas, this article took rural residents as the study sample, investigating the rural neighborhood environment from the two perspectives of the physical and social environment, and further analyzing the relationship between the rural neighborhood environment and residents’ health level. We not only analyzed the self-perceived neighborhood environment of Chinese rural residents that are difficult to be involved in previous studies, but also made a more comprehensive investigation of the association of self-perceived physical and social neighborhood environment with the health of Chinese rural residents. This article supplemented the previous lacking of research on this topic in rural areas, which could provide a useful reference for improving health governance in areas with underdeveloped facilities in other countries. 2. We investigated health outcomes from four aspects, namely depressive mood, self-rated health, chronic disease and BMI, and investigated health-related behaviors from four aspects, that is, time sleeping weekday, physical exercise and tobacco use and alcohol consumption. We analyzed the relationship between rural neighborhood environment and every health status index and health-related behavior index, and thus coming to a more comprehensive conclusion, and deepening the understanding of the relationship between neighborhood environment and health from the social support perspective.

## 2. Materials and Methods

### 2.1. Data Source

The data are originated from the China Family Panel Studies (CFPS) survey. It is a nationally representative survey of Chinese communities, families and individuals launched in 2010 by the Institute of Social Science Survey (ISSS) of Peking University, China. Interviews will be conducted using computer-assisted personal interviewing (CAPI) technology provided by the Survey Research Center (SRC) at the University of Michigan. In the 2016 survey, new items about neighborhood environment have been added, so this article only applies the data of 2016. The study was approved by the Institute of Social Science Survey (ISSS) of Peking University, China.

### 2.2. Study Population

**Figure 1:**
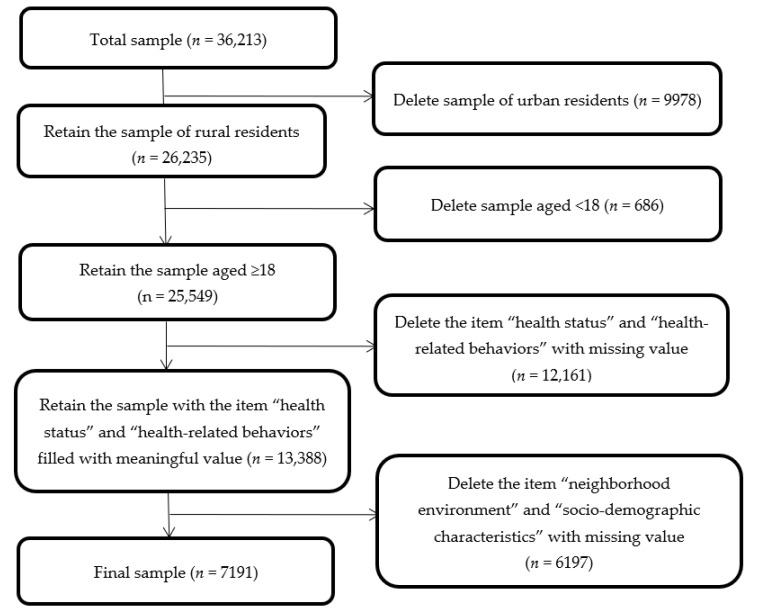
Study sample selection process.

The study population consisted of Chinese adult rural residents who participated in the CFPS 2016, reported their neighborhood environment, health status and health-related behaviors. After excluding those whose data were missing (Figure 1), the study population consisted of 7191 residents.

### 2.3. Measures

#### 2.3.1. Health Status

The assessment of health status includes four variables extracted from the CFPS 2016 questionnaire. They are (1) depressive mood; (2) self-rated health; (3) chronic disease and (4) BMI.

Depressive Mood was assessed by the eight-item version of the Center for Epidemiologic Studies Depression Scale (CES-D8), which is a simplified version of the CES-D20 scale [38,39,40]. In the CES-D8 scale, participants were asked: “Did you experience any of the listed situations for last week”, which includes (1) feeling depressed, (2) feeling strenuous to do anything, (3) suffering from insomnia or hypersomnia symptoms, (4) feeling pleasant, (5) feeling lonely, (6) feeling happy, (7) feeling sad, (8) feeling inability to continue life. Questions are measured by using a 4-point Likert scale (0 = less than 1 day, 1 = 1–2 days, 2 = 3–4 days, and 3 = 5–7 days). The 2 questions assessing positive feelings (“feeling pleasant”, “feeling happy”) were reverse scored. Scale scores are assessed using a non-weighted summated rating and ranged from 0 to 24, with higher scores indicating a higher intensity of depressive complaints.

Self-rated health was measured by the question “What do you think of your health …?” with response categories of “excellent”, “very good”, “good”, “fair” and “poor”. Individuals who were reported to be “excellent to fair” were classified as those who had good health status.

Measures of chronic diseases were derived based on responses to the following questions “Have you had any chronic diseases diagnosed by your doctor in the past six months?” with response categories of “no” and “yes”. These measures have been validated against other measures of health status [41,42] and used previously in several studies [43,44,45].

BMI was calculated by dividing the weight (in kilograms) by the height squared (in meters). It is a commonly used international standard to measure the degree of body weight and health. The higher the BMI index is, the more obese the body and the greater the threat to health will be.

#### 2.3.2. Health-Related Behaviors

Health-related behaviors were assessed by dichotomizing responses to frequency questions based on health recommendations or usual thresholds retrieved in the literature concerning the following: (1) time sleeping weekday; (2) physical activity; (3) tobacco use and (4) alcohol consumption.

Time sleeping weekday was measured by the following questions: “In general, how many hours do you sleep on weekdays?” For the purpose of the present study, frequencies were dichotomized to “<9 h” vs. “≥9 h”.

Physical activity was derived from responses to the following questions: “How many times have you exercised in the past week?”. For the purpose of the present study, frequencies were dichotomized to “none” vs. “at least once”.

With regard to smoking frequencies, the respondents were asked: “Have you smoked in the past month?” with response categories of “no” and “yes”.

Alcohol consumption was derived from responses to the following questions: “Did you drink more than three times a week in the past month?” with response categories of “no” and “yes”.

#### 2.3.3. Neighborhood Environment

The primary independent variable of this study was neighborhood environment. We investigated the physical and social neighborhood environment, respectively. In the study, the physical environment of the community was expressed by the surrounding dwelling environment of the community and the convenience of the community public facilities. The neighborhood social environment was investigated through the situation of public security and neighborhood relationship. Measures of dwelling environment (DE), public facilities convenience (PFC), public security (PS) and neighborhood relationship (NR) were derived from responses to the following question: “What is the surrounding environment (noise pollution, garbage stacking, etc.) of your community?”, “What is the overall situation of education, medical care, transportation and other public facilities around your community?”, “what is the public security situation around your community?” and “In general, what do you think of the neighborhood relationship of your community?”, respectively. Responses to the above four questions were categorized as “excellent”, “very good”, “good”, “fair” and “poor”. Individuals who were reported to be “excellent to good” were classified as those who live in a good surrounding environment.

#### 2.3.4. Socio-Demographic Characteristics

Self-reported demographic data of participants: age (18–59, 60 and above), gender (male and female), marital status (unmarried, married, divorced or widowed), education level (middle school and below, high school/vocational school, college and above) and region (eastern, central, western). The socio-economic status of the residents was measured using the income level, which was measured by the question “What is your level of personal income locally?”, with response categories of “very low, low, fair, high and very high”. Responses were divided into three categories, of which interviewees who were reported to be “very low to low” were classified as whose economic state is poor and those reported to be “high to very high” were classified as whose economic state is fine.

### 2.4. Statistical Analyses

Multivariate ordered logistic regression models were conducted to estimate the strength of association between neighborhood environment and the indicators of depressed mood/self-rated health/time sleeping weekday/physical exercise. Multivariate logistic regression models were conducted to estimate the strength of association between neighborhood environment and the indicators of chronic disease/smoking frequencies/alcohol consumption. A linear regression model was conducted to estimate the strength of the association between neighborhood environment and the indicator of BMI. In addition, variables for potential confounders (age groups in years, gender, marital status, education level, region, household income and social class) were added to the models. Odds ratios (ORs) (with 95% confidence intervals) described the size of the association between the predictor variables and health status/behaviors. All analyses were performed using STATA version 14.0 (STATA Corp, College Station, TX, USA).

## 3. Results

We identified 7191 Chinese rural residents who participated in CFPS2016. Table 1 provides details of the descriptive statistics and sample.

### 3.1. Characteristics of Participants

Among 7191 rural adult residents, 1448 (20.14%) were aged 60 and above, 3870 (53.82%) were males and 3321 (46.18%) were females. Generally, the respondents were not highly educated, with 87.15% of the respondents having a junior middle school education or below. The mean annual household income of the participants reported was 41,516.15 yuan, which was much lower than the average annual household income of 86,358.64 yuan for urban residents in CFPS in the same year. This revealed a significant gap existing between urban areas and rural areas. Nearly a third of the participants (29.69%) considered themselves in a low social class.

### 3.2. Health Status of Participants

The average CES-D8 score for rural residents was 5.32, which was higher than that of urban residents (4.15) researched by CFPS in the same period. In regard to self-rated health, 67.53% of the participants thought they were healthy, with 16.91% thinking their health condition was general and 15.56% thinking they were unhealthy. A total of 15.73% of the participants suffered from chronic diseases, which was higher than 12.44% of urban residents who suffered from chronic diseases. The average BMI of rural residents was 23.28, which was lower than that of urban residents (23.95).

### 3.3. Health-Related Behaviors of Participants

A total of 19.14% of the rural residents slept less than 9 h on weekends, and 67.00% of the rural residents did not take part in physical exercise. Overall, 2402 (33.40%) rural residents were reported being smokers, and 16.27% of the rural residents drank more than three times a week.

### 3.4. Neighborhood Environment Status of Participants

Table 1 showed that 1085 (15.09%) rural residents thought that the DE was poor; 957 (13.31%) rural residents thought the PFC was poor; 584 (8.12%) rural residents thought the PS was bad; and 92 (1.28%) rural residents thought the NR was poor. It can be seen that the majority of rural residents were satisfied with their neighborhood relationship.

### 3.5. The Associations of Self-Perceived Neighborhood Environment with Health Status

Table 2 reported associations between self-perceived neighborhood environment and health status after adjustments for socio-demographic characteristics. Rural residents who reported having a good DE were less likely to report having a depressive mood and poor health conditions. The results showed no significant association between DE and BMI/chronic disease. Rural residents who reported having good PFC were less likely to report having a depressive mood, being unhealthy and having chronic diseases. The BMI of rural residents had no significant correlation with the PFC. Rural residents who reported having good PS were less likely to report having a depressive mood. The self-rated health, chronic disease and BMI of rural residents had no significant correlation with the PS. Rural residents who reported having better NR were less likely to have depressive mood, unhealthy, chronic diseases and obesity.

### 3.6. The Associations between Self-Perceived Neighborhood Environment and Health-Related Behaviors

Table 3 reported the associations between self-perceived neighborhood environment and health-related behaviors after adjustments for socio-demographic characteristics. It was found that DE, PS and NR had no significant correlation with health-related behaviors of rural residents. Rural residents who reported good PFC were more likely to do physical exercise. The time sleeping weekday, smoking frequencies and alcohol consumption of rural residents had no significant correlation with the PFC. It is worth noting that the regression results of control variables showed that higher education levels were significantly correlated with the most beneficial health-related behavior index (more time sleeping weekday, more physical exercise and no smoking).

## 4. Discussion

Health is not only a medical concept, but also a socioeconomic concept. Since socioeconomic differences in health outcomes are possibly avoidable, sufficient attention should be paid on the roles of socio-psychological environment in alleviating health inequalities among residents. The neighborhood environment is individuals’ daily social space. Dwelling environment, public facilities convenience, public security and neighborhood relationship will affect the health of residents. The aim of this article is to analyze the relationship between the neighborhood environment and the health status and health-related behaviors of rural residents of China. Firstly, this article classified the health status of rural residents into depression, self-rated health, chronic diseases and BMI, and then classified the health-related behaviors into time sleeping weekday, physical activity, tobacco use and alcohol consumption. On this basis, we analyzed the relationship between the neighborhood environment and these eight variables.

The conclusions of this article supported the materialist model of health inequality. The materialist model indicated that the impact of exposure to different material factors on residents’ health became more obvious; that is, when facing more environmental risks, health would be more threatened [46].

The results suggested that rural residents who lived in a good neighborhood environment were more likely to report having better health status. If being exposed to air pollution and noise pollution for long-term, or getting access to inadequate facilities for education, medical care, physical exercise or facing security threats in community, rural residents would be in worse mental and physical health [47]. The rural neighbors being acquaintances, and residents in the same region seeing each other almost every day, coupled with the features of small differences and strong cooperation in their occupations, lead to residents not only getting together for entertainment every day, but also having a lot of time to help each other. The neighborhood relationship being better, rural residents were more likely to help each other and communicate with each other more frequently, which is conducive to the search for health information and mutual support. This helps explain why having a better neighborhood relationship tends to reduce depression and improve physical health.

This article further analyzed the relationship between neighborhood environment and health-related behavior of rural residents, and found that among rural residents, the frequencies of taking physical exercise could increase significantly in communities with better public facilities convenience. Complete public facilities of sports could meet the increasingly diversified exercise needs of rural residents, and then attract residents to participate actively in physical exercise [48]. Dwelling environment, public facilities convenience and neighborhood relationship had no significant correlation with health-related behaviors of rural residents, which revealed that health-related behaviors are less affected by neighborhood environment. This is possibly because that health-related behaviors mean healthy lifestyles that relate to individual life habits to a large extent [49]. It is worth to note that the level of education was significantly correlated with each index of health-related behaviors. A study by the University of Pennsylvania shows that people with low education have a higher probability of sleep disorders, which is closely related to their multiple jobs, shift work, noisy living environment, weak social security and low quality of life [50]. We also found that residents with higher education degrees were more likely to drink alcohol. This is because drinking makes it easier to build relationships with colleagues and clients, creating an advantage in building up social networks. In other words, the work environment of highly educated residents requires them to be good at interpersonal communication. It is obvious that drinking is a good way to socialize, and drinking in social situations has a lot to do with excessive drinking [51]. However, at the same time, residents with higher education exercised more actively and were more inclined not to smoke. This can be mainly attributed to the improvement of the level of education, which can not only increase the awareness of disease risks and health information, but also improve the physical exercise values of residents imperceptibly, thus promoting and motivating them to reduce smoking and participate in physical exercise [52].

## 5. Limitations

This article, although is innovative in the research on the association between self-perceived physical and social neighborhood environment and health of Chinese rural residents, it still has limitations: first of all, the neighborhood environment is composed of many elements. In addition to the four indicators involved in this article, neighborhood environment also includes land use and transportation, street design, public space, etc. Limited by data, the study cannot cover other aspects regarding the neighborhood environment. Secondly, in this article, neighborhood environment was reported by rural residents, rather than objective indicators. The advantage of using a subjective index is to investigate the influence of the living environment on physical and mental health from the perspective of residents’ self-feelings. However, because most rural residents do not experience the urban living environment, the self-reported living environment may deviate from the objective situation in the absence of comparison. Thirdly, perceived variables are useful but often lack a robust connection to urban and regional planning policies that aim at modifying the actual neighborhood characteristics. However, CFPS did not report objectively measured neighborhood characteristics. Therefore, in future research, we also need to consider the role of the objectively measured neighborhood characteristics.

## 6. Conclusions

To explore the health status and health-related behaviors of Chinese rural residents and their influencing factors is of great practical significance to improving the health level of rural residents. Based on the 2016 China Family Panel Studies (CFPS) survey, this article suggested that there was strong evidence for a relationship between physical and social neighborhood relationship and the general health of rural residents due to all causes. To be precise, a good neighborhood relationship (including dwelling environment, public facilities convenience, public security and neighborhood relationship) exerted a more significant impact on the health status (including depressive mood, self-rated health, chronic disease and BMI) of rural residents, compared with the relatively limited positive incentive effect on health-related behaviors (including time sleeping weekday, physical activity, smoking and alcohol consumption). This study also found that the level of education was highly related to the health-related behavior of rural residents.

The findings could deepen the understanding of the relationship between neighborhood environment and health from the social support perspective, and provide a useful reference for improving health governance in areas with underdeveloped facilities in other countries. According to the conclusion of this article, in order to improve the health of rural residents, policy interventions should give priority to improving the neighborhood environment. In addition, it is also very important to improve the education level of rural residents, which helps more rural residents to maintain good health-related behaviors.

## Figures and Tables

**Table 1:** Characteristics of the study population.

Variables	Total Population *n* (%)	Variables	Total Population *n* (%)
DE		Household income, mean ± SD	41,516.15 ± 46,411.37
Bad	1085 (15.09)	Subjective Social Class	
Good	6106 (84.91)	Low	2135 (29.69)
PFC		Fair	3356 (46.67)
Bad	957 (13.31)	High	1700 (23.64)
Good	6234 (86.69)	Depressive mood, mean ± SD	5.32 ± 3.96
PS		Self-rated health	
Bad	584 (8.12)	Unhealthy	1119 (15.56)
Good	6607 (91.88)	Fair	1216 (16.91)
NR		Healthy	4856 (67.53)
Bad	92 (1.28)	Chronic	
Good	7099 (98.72)	No	6060 (84.27)
Age groups in years		Yes	1131 (15.73)
<60	5743 (79.86)	Time sleeping weekday	
≥60	1448 (20.14)	<9 h	1376 (19.14)
Gender		≥9 h	5815 (80.86)
Male	3870 (53.82)	Physical exercise	
Female	3321 (46.18)	No	4818 (67.00)
Marital status		At least one time	2373 (33.00)
Unmarried	520 (7.23)	Smoking frequencies	
Married	6386 (88.81)	No	4789 (66.60)
Divorce or bereavement	285 (3.96)	Yes	2402 (33.40)
Education level		Alcohol consumption	
Middle school and below	6267 (87.15)	No	6021 (83.73)
High school/vocational school	646 (8.98)	Yes	1170 (16.27)
College and above	278 (3.87)	BMI, mean ± SD	23.28 ± 3.43
Region		Underweight	442 (6.15)
Eastern	2854 (39.69)	Normal	3929 (54.64)
Central	1533 (21.32)	Overweight	2205 (30.66)
Western	2803 (38.98)	Obesity	615 (8.55)

Note: Abbreviations: DE, dwelling environment; PFC, public facilities convenience; PS, public security; NR, neighborhood relationship.

**Table 2:** The associations between self-perceived neighborhood environment and health status.

Variables	Depressive Mood		Self-Rated Health		Chronic Disease		BMI	
	OR	95% CI	OR	95% CI	OR	95% CI	Coefficient	95% CI
DE (ref: bad)	0.74	0.65–0.84 ***	1.32	1.14–1.53 ***	0.90	0.74–1.09	0.08	−0.14–0.31
PFC (ref: bad)	0.81	0.71–0.92 **	1.30	1.11–1.51 ***	0.79	0.65–0.96 *	−0.04	−0.28–0.19
PS (ref: bad)	0.75	0.64–0.89 ***	1.15	0.96–1.38	0.82	0.65–1.04	−0.17	−0.47–0.12
NR (ref: bad)	0.46	0.31–0.66 ***	2.14	1.42–3.21 ***	0.53	0.33–0.87 *	−1.07	−1.76–−0.38 **
Age (ref: <60)	1.06	0.95–1.18	0.56	0.50–0.64 ***	2.00	1.72–2.33 ***	−1.17	−1.37–−0.96 ***
Gender (ref: male)	1.52	1.40–1.65 ***	0.65	0.59–0.72 ***	1.40	1.23–1.60 ***	−0.25	−0.41–−0.10 ***
Marital status (ref: unmarried)								
Married	0.97	0.82–1.14	0.31	0.23–0.41 ***	4.26	2.64–6.87 ***	1.22	0.90–1.53 ***
Divorce or bereavement	2.18	1.67–2.85 ***	0.28	0.19–0.41 ***	4.52	2.58–7.91 ***	1.27	0.77–1.78 ***
Education level (ref: Middle school and below)								
High school/vocational school	0.86	0.74–0.99b *	1.43	1.18–1.75 ***	0.94	0.73–1.21	−0.20	−0.47–0.07
College and above	0.72	0.58–0.89 **	1.31	0.94–1.84	0.98	0.63–1.52	−0.24	−0.67–0.17
Region (ref: Eastern)								
Central	1.03	0.93–1.15	1.00	0.87–1.14	1.34	1.13–1.60 ***	−0.32	−0.52–−0.11 **
Western	1.69	1.54–1.85 ***	0.81	0.72–0.91 ***	1.50	1.29–1.75***	−1.39	−1.57–−1.22 ***
Social position (ref: low)								
Fair	0.64	0.58–0.70 ***	1.32	1.18–1.48 ***	1.00	0.85–1.17	0.21	0.03–0.39 *
High	0.64	0.57–0.72 ***	1.45	1.26–1.66 ***	1.16	0.97–1.38	0.34	0.12–0.55 **
Household income	0.88	0.85–0.91 ***	1.12	1.08–1.16 ***	0.99	0.95–1.04	0.09	0.03–0.15 **

Note: * *p* < 0.05; ** *p* < 0.01; *** *p* < 0.001; Abbreviations: DE, dwelling environment; PFC, public facilities convenience; PS, public security; NR, neighborhood relationship; OR, odds ratio; CI, confidence interval.

**Table 3:** The associations between of self-perceived neighborhood environment and with health-related behaviors.

Variables	Time Sleeping Weekday		Physical Exercise		Smoking Frequencies		Alcohol	
	OR	95% CI	OR	95% CI	OR	95% CI	OR	95% CI
DE (ref: bad)	1.09	0.91–1.30	0.93	0.79–1.08	0.95	0.79–1.15	1.20	0.97–1.47
PFC (ref: bad)	1.05	0.87–1.27	1.20	1.02–1.42 *	0.95	0.78–1.15	0.81	0.65–1.01
PS (ref: bad)	1.00	0.80–1.26	1.05	0.86–1.28	0.93	0.72–1.18	0.96	0.73–1.27
NR (ref: bad)	0.98	0.57–1.68	1.54	0.93–2.53	1.24	0.70–2.20	1.01	0.52–1.94
Age (ref: <60)	0.77	0.67–0.90 ***	1.32	1.16–1.51 ***	0.94	0.81–1.10	0.98	0.83–1.16
Gender (ref: male)	1.01	0.90–1.15	1.04	0.94–1.15	0.01	0.01–0.02 ***	23.26	17.87–30.26 ***
Marital status (ref: unmarried)								
Married	0.72	0.54–0.95 *	0.81	0.66–1.00	0.81	0.64–1.02	0.72	0.54–0.95 *
Divorce or bereavement	0.53	0.36–0.79 **	0.73	0.53–1.02	0.78	0.52–1.15	0.82	0.52–1.30
Education level (ref: Middle school and below)								
High school/vocational school	1.18	0.94–1.47	1.60	1.35–1.90 ***	0.78	0.64–0.95 *	1.28	1.02–1.61 *
College and above	1.59	1.08–2.34 *	2.95	2.27–3.85 ***	0.31	0.22–0.43 ***	3.36	2.06–5.46 ***
Region (ref: Eastern)								
Central	1.03	0.89–1.20	1.13	0.99–1.29	1.07	0.91–1.27	1.62	1.35–1.93 ***
Western	1.65	1.44–1.90 ***	0.82	0.73–0.92 ***	1.12	0.97–1.28	2.48	2.11–2.91 ***
Social class (ref: low)								
Fair	1.01	0.87–1.17	1.30	1.15–1.47 ***	1.10	0.95–1.28	0.93	0.79–1.09
High	0.77	0.65–0.91 **	1.82	1.58–2.09 ***	1.19	1.00–1.42 *	0.79	0.65–0.96 *
Household income	0.97	0.93–1.02	1.05	1.01–1.09 *	0.96	0.92–1.01	0.94	0.88–0.99 *

Note: * *p* < 0.05; ** *p* < 0.01; *** *p* < 0.001; Abbreviations: DE, dwelling environment; PFC, public facilities convenience; PS, public security; NR, neighborhood relationship; OR, odds ratio; CI, confidence interval.

## Data Availability

Publicly available datasets were analyzed in this study. This data can be found here: https://opendata.pku.edu.cn/dataset.xhtml?persistentId=doi:10.18170/DVN/45LCSO (accessed on 4 August 2021).
